# Draft Genome Sequence of the Antarctic Polyextremophile *Nesterenkonia* sp. Strain AN1

**DOI:** 10.1128/genomeA.00197-14

**Published:** 2014-03-27

**Authors:** Habibu Aliyu, Pieter De Maayer, Jasper Rees, Marla Tuffin, Don A. Cowan

**Affiliations:** a Center for Microbial Ecology and Genomics, University of Pretoria, Pretoria, South Africa; b Biotechnology Platform, Agricultural Research Council, Onderstepoort, South Africa; c Institute for Microbial Biotechnology and Metagenomics (IMBM), University of the Western Cape, Cape Town, South Africa

## Abstract

*Nesterenkonia* sp. strain AN1 was isolated from Antarctic soil and is a polyextremophile, being tolerant of low temperatures, high salt concentrations, and high alkalinity. Here we report the draft genome sequence of this strain.

## GENOME ANNOUNCEMENT

Antarctic desert soil represents one of the most extreme terrestrial environments on Earth (1, 2). Microorganisms surviving in this environment therefore possess several adaptive mechanisms to cope with the low temperatures, elevated pH, salt, high UV irradiation levels, and low water content typical of this harsh environment (3). Members of the genus *Nesterenkonia* are Gram positive, non-spore forming, chemoorganotrophic, aerobic, and moderately haloalkaliphilic (4–6). *Nesterenkonia* sp. strain AN1 was isolated from Antarctic desert soil and represents the first reported psychrophilic representative of the genus (7). A unique aliphatic amidase active on short-chain amides was isolated from the strain (7), highlighting its potential as a source for the identification of novel cold-adapted gene products.

The genome of *Nesterenkonia* sp. AN1 was sequenced using the Illumina GAIIx (5,177,635 reads; mean length, 44 bp; ~36× coverage) and the Ion Torrent PGM (3,842,066 reads; mean length, 324 bp; ~351× coverage) platforms. The reads were *de novo* assembled using CLC Genomics Workbench v. 6, Velvet v 1.2.10 (8), and the DNAStar Seqman NGen assembler v 11. The resultant contigs were further assembled using *in silico* gap closure and reference-based assembly applications, including Mauve (9) and BioEdit (10). The draft genome comprises 42 contigs, with an average size of 72,384 nucleotides, yielding a genome of ~3.05 megabases in size, with a mean G+C content of 67.4%. These results are similar to the sizes (2.59 to 2.81 Mb) and G+C contents (62.2 to 71.5%) observed in the draft genomes of three temperate strains of *Nesterenkonia* which are publically available (11). The genome was annotated using the Rapid Annotations using Subsystems Technology (RAST) server (12). The genome codes for a predicted 2,847 proteins, including 2,159 nonhypothetical and 688 hypothetical proteins, as well as 49 tRNAs.

Currently, we are comparing the draft genome of *Nesterenkonia* sp. AN1 with those of three temperate representatives of the genus for which the genomes have been sequenced (11). These analyses will allow us to elucidate the molecular mechanisms underlying the cold adaptation of this bacterium and identify potential novel biotechnologically relevant cold-adapted enzymes, as well as gain an understanding of the halo- and alkali tolerance typical of the genus.

### Nucleotide sequence accession numbers.

This whole-genome shotgun project has been deposited at DDBJ/EMBL/GenBank under the accession number JEMO00000000. The version described in this paper is version JEMO01000000.
